# In Vitro and In Vivo Models for Evaluating the Oral Toxicity of Nanomedicines

**DOI:** 10.3390/nano10112177

**Published:** 2020-10-31

**Authors:** Sudeep Lama, Olivier Merlin-Zhang, Chunhua Yang

**Affiliations:** Center for Diagnostics and Therapeutics, Digestive Disease Research Group, Institute for Biomedical Sciences, Petite Science Center, Suite 754, 100 Piedmont Ave SE, Georgia State University, Atlanta, GA 30303, USA; slama1@gsu.edu (S.L.); omerlinzhang@student.gsu.edu (O.M.-Z.)

**Keywords:** nanomedicine, oral toxicity, in vitro model, in vivo model, digestive diseases

## Abstract

Toxicity studies for conventional oral drug formulations are standardized and well documented, as required by the guidelines of administrative agencies such as the US Food & Drug Administration (FDA), the European Medicines Agency (EMA) or European Medicines Evaluation Agency (EMEA), and the Japanese Pharmaceuticals and Medical Devices Agency (PMDA). Researchers tend to extrapolate these standardized protocols to evaluate nanoformulations (NFs) because standard nanotoxicity protocols are still lacking in nonclinical studies for testing orally delivered NFs. However, such strategies have generated many inconsistent results because they do not account for the specific physicochemical properties of nanomedicines. Due to their tiny size, accumulated surface charge and tension, sizeable surface-area-to-volume ratio, and high chemical/structural complexity, orally delivered NFs may generate severe topical toxicities to the gastrointestinal tract and metabolic organs, including the liver and kidney. Such toxicities involve immune responses that reflect different mechanisms than those triggered by conventional formulations. Herein, we briefly analyze the potential oral toxicity mechanisms of NFs and describe recently reported in vitro and in vivo models that attempt to address the specific oral toxicity of nanomedicines. We also discuss approaches that may be used to develop nontoxic NFs for oral drug delivery.

## 1. Introduction

Oral administration is one of the most popular routes for delivering drug formulations due to its convenience of dosing and excellent patient compliance. While conventional oral formulations are constructed to increase the drug’s systemic bioavailability, nanoformulations (NFs) are often fabricated to enable the precise release of the encapsulated drug to the disease site by active targeting (including ligand-receptor mediated and antibody-, pH-, or reactive oxygen species (ROS)-responsive targeting) and/or passive targeting (such as through the enhanced permeability and retention (EPR) effect) approaches [1,2,3,4]. Therefore, NFs can reduce the systemic side effects of a loaded drug by decreasing the systemic drug biodistribution into normal tissues and increasing the drug biodistribution into the diseased tissue. Oral NFs are also designed to stabilize the encapsulated drug in the harsh gastrointestinal (GI) environment and locally release it in a controlled or sustained manner [5]. Thus, orally deliverable NFs are believed to be the most promising drug delivery system for fighting various diseases, especially digestive diseases, such as inflammatory bowel disease (IBD) and colon cancer [6,7].

Various materials, including inorganic (metal oxides, silicon dioxide) and organic (polymers, biological macromolecules, and other biocompatible materials) nanomaterials, have been used to fabricate oral NFs [8,9,10,11]. Among them, three types of NFs (nanocrystals, nanoliposomes, and nanopolymers) were successfully marketed, with more than 90% of marketed NFs being made from organic materials [12]. Generally, the toxicity of NFs can originate from pristine NFs, partially degraded NFs, nanocomposite chemicals, or premature release of the loaded drug [13,14,15,16]. As scientists tend to employ biocompatible chemicals to construct NFs and the loaded drug is generally present at a much lower dose compared to the conventional formulations [17], current toxicity studies mainly focus on the harmful effects that originate from intact and partially degraded NFs.

Intact NFs have unique physicochemical properties, including their size, aspect ratio, microstructure, surface charge, and surface chemistry. These properties determine their in vivo efficacy and toxicity upon oral administration [18,19]. Typically, at least one dimension of an NF is within the nanoscale (between 1–100 nm). Therefore, the surface area to volume ratio (SA/V) of NFs is much larger than that of traditional formulations with the same volume. This vast SA/V elevation greatly increases the interaction between the NFs and GI environment, potentially increasing the toxicity [13,15,20,21]. Studies have shown that nanosized gold (Au) and silica (Si)-nanoparticles (NPs) are much more toxic than normal-sized Au and Si particles, which would significantly reduce the therapeutic index (TI) of the Au- and Si-based nanomedicines [11,21,22]. The aspect ratio (AR) of an NF represents the ratio of the particle’s length to its width. A change in AR affects the NFs’ cellular uptake, biodistribution, and toxicity [23,24]. Although scientists have found that NFs (such as anodic alumina (Al) nanotubes) with broad AR distributions (ranging from 7.8 to 63.3) were associated with uncertain safety and toxicity [23,25,26], it is still challenging to determine the direct relationship between the AR of an NF and its toxicity. Some quantum-sized NFs have extremely high surface energy and reactivity (a massive surface area available for reactions) [27,28]. The high surface energy is often manifested in the formation of free radicals (e.g., ROS) that can damage proteins and DNA [29,30]. However, quantum-sized NFs composed of cerium dioxide (CeO_2_) were found to suppress ROS toxicity, suggesting that ROS generation cannot be predicted solely from the size of NFs [31,32,33]. Indeed, the surface chemistry and composition of NFs have also been found to contribute to their toxicity. Studies have shown that the dissolution of toxic ions, such as zinc (Zn^2+^) and copper (Cu^2+^), from the surface of NFs leads to cellular malfunction [34,35,36]. Moreover, the surface chemistry of an NF determines its surface charge, which is responsible for its colloidal stability inside the GI tract [37,38].

Extreme variations in the biological conditions (such as digestive enzyme concentrations and intestinal microbiota compositions) and chemicals (such as pH value and ionic strength) of the GI tract could affect the stability of intact NFs to facilitate their degradation and trigger premature drug release [39,40]. Partially degraded NFs comprise NP fragments that vary in size, zeta potential, and other properties [41,42,43]. Some NP fragments are within the quantum size range and thus can induce immune toxicity. Catalyst NPs, which are often used to generate synthetic NFs, can be co-encapsulated inside an NF during nanofabrication. Upon degradation of the NFs, the catalyst NPs are released and can cause immune toxicity [44,45,46]. Indeed, in the presence of residual catalyst-NPs, carbon nanotubes presented high variations in the results of batch-to-batch toxicity evaluations [44].

As the toxicity of NFs involves multiple mechanistic layers, we will first briefly review the relevant mechanisms of NF-generated toxicity and then introduce major in vitro and in vivo models that have been established to evaluate the toxicity of NFs. Further, we will detail the observed endpoints used in these models and discuss some strategies for making nontoxic NFs.

## 2. Mechanisms of Oral Nanotoxicity

Upon oral administration, the interactions between NFs and GI tract might result in various toxic effects, causing changes of mucus, epithelial cells, tight junction, and gut microbiota and inducing immune responses [47,48,49,50]. The mechanisms by which the toxicity plays out are complicated and may include the following approaches:

### 2.1. Oxidative Stress, Immune Responses, and Inflammation

**Figure 1:**
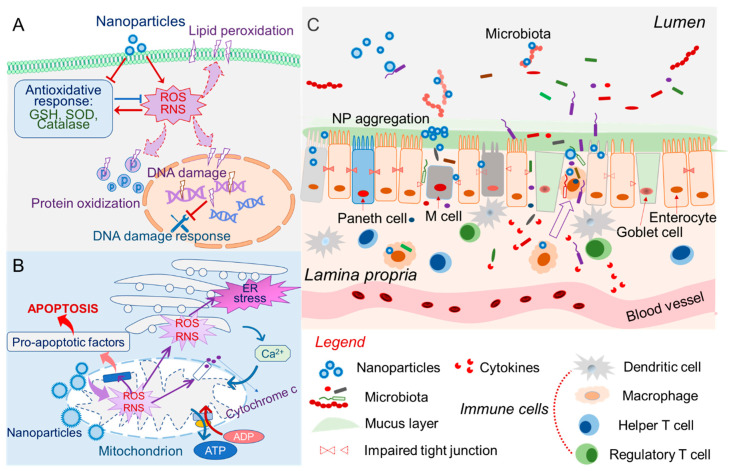
Mechanisms of oral nanotoxicity: (**A**) nanoparticle-induced oxidative stress; (**B**) nanotoxicity of intracellular organelle damages; and (**C**) nanoparticle-induced microbiota abnormality, immune responses, and damages to the gut barrier and luminal environment.

Oxidative stress is caused by an imbalance between the production and elimination of free radicals (including reactive oxygen and nitrogen species (RONS)) [51]. Although elevated free radicals (at a certain level) act as signaling molecules to help maintain physiological functions, excessive accumulation of RONS can damage lipids, proteins, carbohydrates, and DNA [52,53,54,55] (Figure 1A). Such damages can trigger immunogenicity, which is a leading cause of acute and chronic inflammation. The toxicity of NFs can involve actions that either induce excessive production of reactive species or hamper the RONS-eliminating function of the cells; often, such toxicity results from a combination of these processes [56,57] (Figure 1A).

Some nanomaterials present on the NF surface can prompt excessive RONS production. For example, NFs composed of metal oxides may catalyze the formation of oxygen-derived metabolic products, generating intracellular reactive hydroxyl radicals [58]. Hydroxyl radicals further deteriorate the double bonds of unsaturated fatty acids in a process known as lipid peroxidation [59]. Moderate lipid peroxidation causes malfunction of many organelles, including the plasma membrane, mitochondria, endoplasmic reticulum (ER), and lysosome [60,61]. Severe lipid peroxidation can lead to membrane dysfunction (changes in potential and permeability, loss of fluidity) and cell apoptosis [61,62]. Therefore, reactive-species-induced lipid peroxidation is a crucial path to oxidative stress. NF-induced RONS can also trigger oxidative carbonylation in proteins that contain arginine, histidine, lysine, proline, or threonine side chains to form toxic reactive aldehydes or ketones [63,64]. This is one of the most harmful oxidative protein modifications and causes irreversible and unrepairable injury to cells [65]. Further, excessive ROS in the nucleus can induce DNA damage and affect the DNA damage response (DDR) (Figure 1A), causing DNA single-strand breaks and impairing surveillance proteins, leading to gene mismatch and erroneous DNA repair [66]. A high concentration of nuclear ROS may even cause dangerous DNA double-strand breaks (DSBs), which cause mutagenesis via chromosomal rearrangements or missing genetic information [22,30,67].

Cells routinely produce antioxidants, such as reduced glutathione (GSH) and α-lipoic acid, to neutralize excessive ROS and maintain normal functions [68,69]. Cu-based NFs were found to decrease the cellular level of GSH and disturb the mitochondrial transmembrane potential in bone-marrow-derived primary macrophages, suggesting that Cu NPs directly impair the antioxidant-producing functions of macrophages [70]. Upon oral treatment of titanium dioxide (TiO_2_) NPs, a corresponding increase in the micronucleus frequency was observed in hepatic cells, indicating that TiO_2_-NPs are potentially genotoxic [71,72]. This could be attributed to a decrease in the GSH to oxidized glutathione (GSSG) ratio (GSH/GSSG) and concomitant increases in ROS generation and lipid peroxidation. Activation and upregulation of superoxide dismutase (SOD) represent another cellular defense approach against oxidative stress [73]. However, SOD activation can be hampered by a high dosage of zinc oxide (ZnO) NPs: Treatment of rats with 50 or 100 mg/kg ZnO-NPs significantly affected the normal activation of SOD and induced observable intestinal injury [74].

Beyond the cellular redox-pathway, NFs can also directly interact with innate immune cells, such as T-cells, macrophages, and neutrophils [75,76,77,78]. NF-triggered immune responses activate the excessive secretion of proinflammatory cytokines, including TNF-α, interleukin (IL)-1β, IL-6, and IL-8, which can then cause acute inflammatory effects to the body [79,80]. Moreover, lipophilic polymer NFs may interact with and disturb the membrane lipids of cells, which may affect the respiratory chain and ROS signaling [81].

### 2.2. Nanotoxicity of Intracellular Organelles

Accumulated evidence showed that cell-engulfed NFs are enrolled in the endosome–lysosome degradation pathway [82]. NFs with negatively charged or neutral surfaces are trapped in endosomes and delivered to lysosomes; these NFs often cause lysosome membrane permeabilization (LMP) [83,84]. LMP can further induce cytosolic acidification, which initiates the degradation of cellular components and leads to apoptosis [85,86]. NFs with a positively charged surface can generally escape from the endosome–lysosome pathway, so these NFs have been widely used for intracellular drug delivery [82,87]. In some cases, NFs that have escaped the endosome can still induce excessive oxidative stress, inducing damage to other organelles, particularly mitochondria and the ER [88] (Figure 1B). NP-induced mitochondrial damage releases cytochrome C and activates pro-caspase 9, leading to caspase 9 activation and cell apoptosis [89,90]. NFs may also trigger the accumulation of unfolded proteins in the ER, fostering the ER injury [91]. Unfolded proteins activate the so-called “ER stress” pathway [92], which joins the mitochondrial-dependent apoptosis pathway.

The accumulation of excessive NFs can also instigate autophagy-related functions [85,93,94], potentially leading to the accumulation of autophagosomes and activation of a caspase-independent cell death pattern. Together, these mechanisms might lead to autophagic cell death.

### 2.3. Genotoxicity

Genotoxicity occurs via damage to the genetic materials within the nucleus, including chromosomal fragmentation and rearrangement, DNA strand breakages, point mutations, and alterations of gene expressions [95,96,97]. NP-induced genotoxicity can be generally categorized as primary or secondary damages [27,70,98]. Primary damages include the direct and indirect interaction of NFs with genetic materials. Direct primary genotoxicity is mostly caused by interactions between NFs and chromosomes during interphase, with NFs binding to DNA molecules and preventing DNA replication or transcription. NFs can also bind to chromosomes during mitosis, thereby inducing clastogenic or aneugenic effects [99]. Graphene oxide (GO) was found to directly induce DNA damage in the intestinal cells of the *Drosophila* larva, as detected by nuclear staining (Hoechst) and the comet assay. The same authors subsequently reported that the oral administration of GO causes various behavioral and developmental defects in the offspring of *Drosophila* [100]. Indirect primary genotoxicity is typically related to NF-generated ROS or NF-released toxic ions. These toxins interfere with proteins responsible for normal genetic functions, such as DNA replication, transcription, or repair [101,102]. ROS may cause oxidization of purines and pyrimidines, leading to mispairing and consequent destructive mutations. Further, excessive generation of ROS induces secondary genotoxicity and downstream inflammatory responses that involve the activation of phagocytes, such as macrophages and neutrophils [103].

The genotoxicity of NFs is determined by their dosage and properties, including their chemical composition, particle size, shape, surface charge, and solubility. The chemical composition is the primary factor for determining genotoxicity: NFs made from uncoated highly toxic chemicals (such as As_2_O_3_ or CdSe) are undoubtedly toxic, regardless of their size and shape [104,105,106]. In addition, decreasing the sizes of NFs or increasing their SA/V may induce higher ROS production, increasing the possibility of genotoxicity. Today, most primary genotoxicity is addressed by in vitro tests. For instance, when investigating the genotoxic potential of ZnO NPs, most studies only described in vitro assessments of the oxidative gene damage arising from dissolved Zn^2+^ ions [107,108]. In the context of studying secondary genotoxicity, in vivo models are often needed to evaluate inflammatory responses to the NFs [109,110].

### 2.4. Nanotoxicity of Gut Barrier

The gut barrier includes a loosely adherent mucus layer (in the colon), a firmly adherent mucus layer, epithelial cells, and tight junctions. The gut barrier and luminal biochemical environment are two vital parts that play essential roles in the pathways of NF-induced toxicity. Chitosan NPs can absorb luminal cations such as calcium (Ca^2+^) and magnesium (Mg^2+^), and this has been shown to affect the formation of tight junctions [111,112,113]. Therefore, the oral administration of chitosan may compromise the intestinal barrier function. Transepithelial electrical resistance (TEER) measures the resistance across the epithelial monolayer and is a sensitive method to detect the integrity of cell monolayer [114]. The reduction of TEER values is associated with an increase of monolayer permeability, which suggests that the gut barrier experiences tight junction disruption or cell death. In TEER assays evaluating the nanotoxicity of oral NFs, differentiated Caco-2 cell monolayers are widely used [115]. Studies have shown that ultrasmall-size (10 to 15 nm in diameter) metal-NPs (made from iron (Fe) and CuO) decreased the TEER values of Caco-2 monolayers and negatively impacted the integrity of tight junctions, as confirmed by staining for tight junction protein-1 (zonula occludens-1 (ZO-1)) [116,117,118]. Such a result indicates that oral administration of these metal NPs could induce intestinal leakage, leading to immune responses and triggering acute inflammation when the gut barrier function against pathogens becomes compromised. CuO NPs were also found to increase IL-8 production in Caco-2 cells, which may induce acute inflammation via neutrophil recruitment and degranulation [117]. Although many metal NPs were found to carry a risk of destroying the gut barrier, some metal oxide NPs (such as CeO_2_ NPs) have been shown to be exceptionally safe even at a very high dose (up to 200 μg/mL) [33]. In Caco-2-based in vitro assays, CeO_2_ NPs did not disturb monolayer integrity, produce genotoxicity, or trigger oxidative DNA damage. The initial size of such CeO_2_ NPs was about 70.33 ± 49.61 nm, as tested by transmission electron microscopy (TEM), suggesting that large-size metal-NPs might be safer than their ultrasmall-size counterparts.

### 2.5. Nanotoxicity and Luminal Environment

In the context of luminal-environment-involved nanotoxicity, the main mechanism could generally contribute by side effects induced by aggregation or degradation of the NFs by the luminal fluid/content [119]. The extreme changes of pH from acidic (in the stomach) to alkaline (in the intestine) is expected to precipitate an NF via aggregation if it experiences a pH value near its point of zero charge (PZC). For example, silver (Ag) NPs, which are typically designed to disperse at a neutral pH in the buffer, have a PZC around pH 3.0 and thus aggregate in the stomach of tested animals [120,121]. Furthermore, the strongly acidic stomach fluid often degrades metal NPs (such as Cu-based NFs and CdTe quantum dots) to release toxic trace metals [122]. To increase biocompatibility, researchers often decorate the surfaces of metal NPs with natural organic compounds, such as a lipid or polypeptide [123,124]. These organic coatings are prone to undergo degradation in the stomach luminal fluid, which is strongly acidic and contains various enzymes; this can alter the surface chemistry and locally precipitate the NFs, potentially inducing an acute immune response.

Beyond the strong pH of luminal fluids, a high concentration of ions (mainly H^+^, Na^+^, K^+^, Ca^2+^, and Cl^-^) can also affect the colloidal stability of NFs. For example, Gitrowski et al. reported that TiO_2_ NPs exhibited accelerated particle settling in the gut saline (within 4 h), leading to excessive exposure of TiO_2_ NPs to the underlying tissue [125]. Because of the high ionic strength and strongly acidic pH, simulated gastric fluid (SGF) was found to be able to agglomerate various nanomaterials, including those containing SiO_2_, Fe_3_O_4_, and Ag [126]. The luminal fluid of the small intestine also contains numerous enzymes, such as pepsin, trypsin, carboxypeptidases, lipase, and nuclease. These enzymes can typically degrade protein- and lipid-based NFs and might even degrade the polypeptide or lipid surface coatings of NFs [127].

### 2.6. Nanotoxicity Involves Microbiota

The mammalian gut lumen is an anoxic environment that hosts trillions of commensal microbes [128]. Most gut microbe species are anaerobic, which helps maintain a healthy reducing biochemical microenvironment [129,130]. The gut microbiota consumes hard-to-digest carbon sources from food and produces essential metabolites, such as short-chain fatty acids (SCFAs) and vitamins, thereby balancing the biochemical composition of the luminal fluid in a way that benefits both the microbiota and the host [131]. An abnormal gut microbial structure is associated with various metabolic and immune diseases, such as obesity, metabolic symptoms, and inflammatory bowel disease (IBD) [132,133]. Studies have suggested that many NFs can shift the healthy microbial structure towards an abnormal one, suggesting that the gut microbiota lands on the footpath that connects NFs to cytotoxicity [76] (Figure 1C). Carbon-based NFs, such as graphene nanosheets, can form noncovalent interactions (mainly π–π stacking, hydrophobic, and electrostatic interactions) with the membranes of Gram-positive bacteria (GPB), thereby damaging the membranes of these bacteria [134,135]. Gram-negative bacteria (GNB) can tolerate graphene-induced membrane damage, and graphene NPs were found to decrease the GPB/GNB ratio, inducing subsequent toxicity. Metal NPs, such as Ag NPs, were reported to reduce GPB, such as *Firmicutes* and *Lactobacillus*, to significantly enhance the proportion of GNB in rats, leading to microbiota-associated immune toxicity [136,137]. In another study, Ag NPs decreased the proportion of coliform bacteria in the intestine of piglets and increased their subsequent chance of overweight [138,139]. In addition to their influence on the microbiota structure, smaller-size Ag-NPs (~20 nm) were found to significantly reduce the diversity of gut microbiota in *Drosophila* [19]. Although it is unclear how the physiochemical properties of NFs directly connect to their biological effects on the microbiota, gut microbial structure and diversity undoubtedly play an essential role in mediating NF-induced changes in the luminal environment [49].

As we continue to discover the mechanisms through which NFs interact with biological systems, we are also learning about their potential toxicity, which may result in serious adverse effects in late-stage trials. This is a serious issue in clinical settings, where NFs are increasingly being used as therapeutics or as drug delivery systems. Thus, there is an urgent need for researchers to develop models that may be used to assess nanotoxicity in a preclinical setting.

## 3. In Vitro Toxicity Models for Orally Delivered NFs

Upon oral administration, NFs interact with different biocompartments, such as the esophagus, stomach, small intestine, and large intestine [140,141]. Each compartment presents distinct physiological conditions, with significant variations in the pH, enzymes, pressure, and surface structure. This challenges the NF structure, and NFs may lose efficacy and/or increase in toxicity as they move along the digestive process. For example, polystyrene NPs exposed to in vitro digestion experienced protein corona changes [142], and their potential to cross gut epithelium cells was enhanced in a culture model. 

### 3.1. Static and Dynamic Culture Models

**Figure 2:**
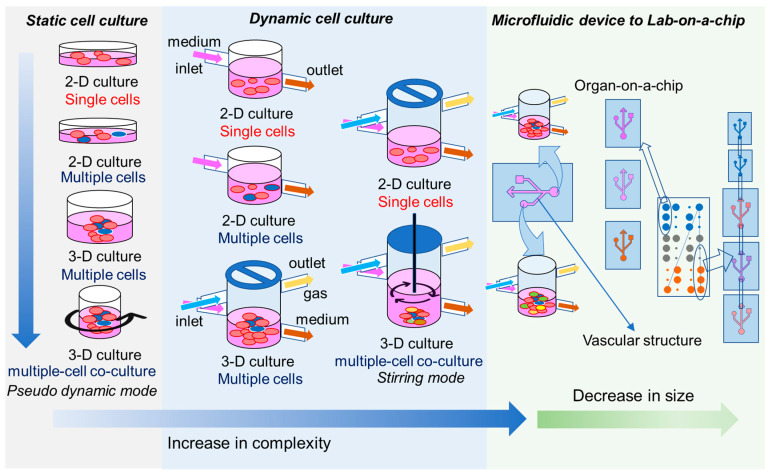
Evolution of in vitro toxicity models.

In vitro digestive models are generally classified as static or dynamic models (Figure 2). In a static gut cell culture model, the culture medium is replaced at a scheduled time interval; in contrast, dynamic models employ continuous movement of fluid to nurture the cells [11,143,144,145,146,147]. In recent years, the in vitro digestive models have tended to increase in their complexity. The selection of cultured cells moved from simple undifferentiated epithelial cells (such as Caco-2 cells) to multiple-cell coculture systems (such as Caco-2/HT29/Raji B) [148] (Figure 2), and the culture medium has evolved from a mixture of essential nutrients and growth factors to synthetic biomimetic fluids with a composition very close to those of the relevant in vivo biofluids [34,149]. The evolution of these models has enabled researchers to better simulate a microenvironment that is closer to the real digestive system. A key consideration in these assays is the choice of cell type. Although Caco-2 cells have been widely used for in vitro intestinal models, the use of human stem cells for in vitro studies may be more rational given that stem cells play critical roles in maintaining the homeostasis of the intestinal environment, such as by modulating cell differentiation, immune responses, wound healing, and host–microbiota interactions [150,151,152,153].

Both static and dynamic digestive models can be operated in a high-throughput fashion, offering a powerful tool for screening large varieties of NFs before in vivo oral testing is performed [154,155]. The composition of synthetic digestive fluid can be changed to simulate different physiological conditions (such as fed vs. fasted) or different age groups (young vs. adult) [144,156], expanding their applications in different disease scenarios. To date, researchers have successfully employed two-dimensional (2D) cell cultures for most high-throughput digestive in vitro models [154]. However, 2D cell growth does not fully represent the real in vivo situation of the cells, as it commonly fails to maintain the cells’ differentiated functions.

### 3.2. 3D Cell Culture Models

Cells have been shown to behave more naturally when cultured in a three-dimensional (3D) system [157,158]. In 3D culture models, intestinal cells have been embedded in a 3D extracellular matrix (ECM), which can increase cell differentiation and reduce cell proliferation [153,159]. This strategy improved the apicobasal polarization and lumen formation of intestinal epithelial cells. However, 3D models are not without their limitations: they lack tissue vasculature, and nutrient access is limited to simple diffusion and can be affected by the gradient of cells [160,161]. Moreover, changes in cell behavior may be subject to geometry and can have far-reaching impacts on the toxicological response. To solve these problems, researchers employed microfluidic technologies in combination with 3D culture, creating miniaturized tissue (or organ) models that offer a more precise and controllable cellular microenvironment [162] (Figure 2). These sophisticated systems have proven to be capable of offering in vivo like cellular microenvironments that can provide nutrients and specific stimuli via controllable gradients [163]. Although studies have suggested that 3D systems should be applied whenever possible, it has proven difficult to establish a universal 3D platform for testing various types of orally delivered NFs [159]. 2D cell culture approaches are still widely used in nanotoxicity studies and can well recapitulate several in vivo behaviors. 3D platforms currently provide an attractive alternative for 2D cell culture for nanotoxicity tests, and they are likely to replace many 2D models once the technology develops to the point that the 3D systems are similar to the 2D systems in terms of their stability and reproducibility.

As a new strategy in 3D culture, researchers adopted microfabrication technologies from the microchip industry to form a new branch termed organ-on-a-chip (OOAC) [164,165]. The OOAC approach creates a microenvironment that includes new features, such as spatiotemporal nutrition gradients, tissue–tissue interactions, and mechanical forces reminiscent of those found in living organs. These features have potentiated the development of new in vitro disease models that can mimic the human in vivo physiology in an organ-specific fashion [165,166]. The gut-on-a-chip (GOAC) model includes in vivo like structural features, such as peristalsis and villi, and contains functional cells such as enterocytes, goblet cells, and endothelial cells [167,168,169]. Importantly, these models can be operated in a high-throughput manner and are compatible with modern bioanalytic systems, such as those used for chromatographic, electrophoretic, and flow cytometry analyses [170,171]. Given its reduced requirements in terms of cell number, culture media, and reagents, the GOAC assay offers a low-cost but still highly reproducible toxicity screening platform for orally administered NFs [171,172,173]. GOAC may partially replace several animal models used in drug development and customized toxicology studies.

### 3.3. Models that Involve the Gut Microbiome

The in vitro microbiome model is a valuable tool for studying indigenous gut microbiota and the therapeutic or adverse effect of NFs that may have antimicrobial functions [174]. It has proven challenging to establish a useful in vitro microbiome model, as researchers have struggled to replicate the real complex living environment of the gut microbiota. Some useful in vitro models have been commercialized, such as ProDigest’s simulator of the human intestinal microbial ecosystem (SHIME) and TNO’s computer-controlled in vitro model of the colon (TIM-2) system [175,176,177]. SHIME is a six-stage reactor system that operates in a continuous culture condition to mimic the in vivo human gastrointestinal tract [178]. Recently, ProDigest developed Mucus-SHIME (M-SHIME), a new model that integrates the mucosal compartment in the colonic mimetic regions of the SHIME and allows the microbiota to adhere to the gut mucus layer under representative conditions [179,180,181]. TNO’s TIM-2 system is an in vitro colon system that was initially developed to study the behavior of genetically modified bifidobacteria and their role in the colon; since then, it has been widely used for in vitro gut microbiota studies [182,183,184]. These commercial in vitro models offer a controllable and reliable platform for studying the impact of NFs on gut microbiota.

Most researchers have treated the entire microbiota community and used commercialized platforms to monitor the effects of such treatment [185,186]. However, some results have been inconclusive and/or conflicting because the complicated responses of the microbiota to such treatments are sometimes beyond the capability of established models [187]. Accordingly, some researchers sought to simplify the microbial community to increase reproducibility. In the simplified human intestinal microbiota (SIHUMI) model [188], for example, the complex microbial community was represented by only seven bacterial species (*Anaerostipes caccae*, *Bacteroides thetaiotaomicron*, *Bifidobacterium longum*, *Blautia producta*, *Clostridium ramosum*, *Escherichia coli*, and *Lactobacillus plantarum*) [188,189]. Although SIHUMI was developed on the germ-free rat model, it is a practical approach for in vitro use because the SIHUMI community was monitored over time by a variety of detection methods, including flow cytometry, intact protein profiling, terminal restriction fragment length polymorphism analysis, and untargeted metabolomics [190,191]. These bioanalytic assays covered both the cellular and molecular levels and offered strong evidence for the robustness of the SIHUMI species. It could thus be an excellent alternative model for studying the nanotoxicity of NFs toward the microbiota.

A limitation of the above-mentioned in vitro models is the lack of information regarding microbiota-mediated immune responses. The newly developed microfluidics-based human–microbial crosstalk model (HuMiX) may improve this situation [192]; HuMiX allows the coculture of human intestinal cells and microbiota under anaerobic conditions that mimic the gastrointestinal human–microbe interface. Importantly, HuMiX recapitulated in vivo immunological responses in human intestinal epithelial cells after coculture with the commensal *Lactobacillus rhamnosus* GG (LGG) [193]. Other in vivo features, such as transcriptional responses, were also found in the coculture of human epithelial cells with the obligate anaerobe *Bacteroides caccae* and LGG. Although HuMiX has not been applied to study the immune response associated with oral nanotoxicity, it may offer a useful tool for such fundamental studies.

### 3.4. Supplementary Test for Determining NP Induced Mutagenicity

The Ames test, which is known as the reverse mutation assay, uses bacteria to assess the mutagenicity of a tested sample [194]. Specifically, several strains of the bacterium *Salmonella Typhimurium* are used, allowing the detection of different mutation types. In general, the bacteria require histidine for growth but cannot produce it. The formation of colonies in the presence of an experimental NF but the absence of histidine would indicate that there has been a reverse mutation in the histidine locus, allowing the bacteria to regain their ability to synthesize histidine [195]. A positive result from Ames assay suggests that the tested NF might be a carcinogen, but Ames should only be used as a supplementary test and not as a stand-alone. Further, in vivo tests are required for confirming the mutagenicity.

## 4. In Vivo Models

Preclinical evaluation of in vivo toxicity is a standardized process; it is usually conducted following good laboratory practices (GLPs) since most of these formulations are intended for clinical trials [196,197]. Such evaluations include assessments of the maximum tolerated dose (MTD), maximum feasible dose (MFD), acute toxicity, and chronic toxicity and should be tested in both rodent and nonrodent animal models [198,199,200]. However, we are herein mainly focused on the discovery phase of nanomedicine studies, where customized models have been established to evaluate the toxicity of various novel NFs. Although discovery-stage nanotoxicity studies still follow the general basics of such standardized processes, they are generally more flexible and may involve the application of innovative nonstandardized models.

### 4.1. Invertebrates

Invertebrate models are useful tools for investigating fundamental toxicology questions and generally present minimum ethical obstacles. They also have many advantages compared to widely used rodent models, such as lower cost, ease of practice, and shorter experiment cycles (because of their short lifespan). *Caenorhabditis elegans* is an excellent example of studying the oral toxicity of NFs [201]. It is convenient for researchers to monitor fluorescent-labeled lipid-core NFs or cadmium telluride quantum dots (CdTeQD) as they travel along the GI tract upon oral administration [202,203]. Live imaging of *C. elegans* provided indisputable evidence of the migration path of CdTeQD via the intestine to the reproductive system, which is the accumulation/toxicity site of CdTeQD. Thus, this model is especially useful for studying the accumulation target and the chronic toxicity of NFs [203].

*Drosophila melanogaster* represents another excellent model in which researchers can initiate research on the toxicity (especially immunotoxicity) of NFs, since the innate immunity of *Drosophila* has been extensively studied [204,205,206]. The different developmental stages of *Drosophila* (i.e., egg, embryo, larva, pupa, adult fly) enable researchers to assess the stage-specific and long-term toxic effects of NFs. S. Priyadarsini et al. studied the toxic impact of graphene oxide (GO) nanosheets on the growth of *Drosophila*. Flies were fed GO-mixed food, and the different developmental stages of the offspring were checked for morphology, behavior, and gene defects [100]. GO affected the crawling speed and trailing path and induced DNA damage in the gut cells of the larva. GO was also associated with phenotypic injuries to the adult fly, with defects observed in the eyes, mouthparts, thorax bristles, and wings. This study exemplifies that *Drosophila* is a practical platform to evaluate the toxicity of orally administrated NFs. Researchers have also established different *Drosophila* autophagy transgenic lines because autophagy genes and encoding proteins are structurally conserved between *Drosophila* and humans [93]. These transgenic models are readily available to study the autophagic effects of NFs.

### 4.2. Zebrafish (*Danio rerio*)

The zebrafish’s intestinal system is similar to that of humans, and its genes share more than 70% similarity with those of humans [207,208]. Such features make it a feasible model for studying oral toxicity. Moreover, the zebrafish has a high reproduction rate and can be inexpensively maintained, thus permitting large-scale experiments. With the available genetic tools, transgenic zebrafish can be easily used to study the toxicological mechanisms of orally administrated NFs. Employing a zebrafish model, J.S. Choi demonstrated that ZnO-based NFs affected the gene expression levels of aicda, cyb5d1, edar, intl2, ogfrl2, and tnfsf13b, which were shown to be related to inflammation and immune responses [110]. Such a genotoxic effect caused yolk-sac and pericardial edema in the embryonic/larval developmental stages of the zebrafish. In another study, Si NPs were found to lower the blood flow and blood velocity in zebrafish embryos [209]. Si NPs trigger inflammatory responses via neutrophils and damage vascular endothelial cells. In a study of microbiota-related nanotoxicity, D.L. Merrifield et al. revealed that Ag NPs influence the diversity and population of the microbiota in zebrafish and that these effects are particularly strong in males [210]. Similar to *Drosophila*, several transgenic zebrafish models are available for studying autophagy-induced toxicity [93,211,212]. Indeed, the zebrafish might be an ideal model for the large-scale detection of NF-induced autophagy. Granted, the high-efficiency, high-throughput screening of hazardous NFs using zebrafish models should not be viewed as a replacement for traditional in vivo analyses in rodents and higher organisms. However, it holds an important place as an intermediate screening assay, between in vitro toxicity screening and the use of higher organisms. Indeed, the zebrafish is a useful platform for predetermining the toxicological mechanisms that may be triggered by orally delivered NFs.

### 4.3. Rodent Models

Most of the in vivo toxicity research on NFs has been carried out using rodent models, mainly rats and mice, with limited studies performed in hamsters. The beauty of using a rodent model for studying oral nanotoxicity is that rodents have digestive and circulation systems similar to those of humans, enabling researchers to accurately extrapolate the results of hematology tests from rodents to humans [213,214,215]. It is convenient to practice toxicodynamic and pharmacodynamic studies using blood drug concentration data obtained from rodents [216,217]. It is also relatively easy to perform endoscopic and histopathologic analyses of the intestine of rodents. For these reasons, rodent models have been widely used as standardized procedures to evaluate the toxicity of formulations, including NFs, and are well-documented in the FDA and EMA guidelines [199,218]. This review will not focus on these standardized procedures; rather, we emphasize that NFs do exhibit distinct species-dependent toxicities even between rodent models (mouse and rat) [219]. Thus, researchers should use caution when extrapolating the toxicology parameters between different animal models.

### 4.4. Nonhuman Primate Models

While rodents are helpful in answering most toxicological questions, their functionality is limited by their less-developed immune systems and different metabolism functions compared to primates [215,220]. The most significant advantage of using nonhuman primate models is that these models can provide essential clues about behavioral changes in the nanotoxicity experimental group. However, due to the difficulty of handling, the need for physical restraints during dosing, and controversial ethical problems, only a few toxicological nonhuman primate models have been established. According to K.T. Yong’s report, the rhesus macaque is the most widely used primate for nanotoxicity research [221]. These authors also performed the first primate toxicity assessment of a quantum dot (QD), intravenously injecting 25 mg/kg of phospholipid-micelle-encapsulated CdSe-Cds-ZnS QDs to male rhesus macaques [222]. In addition to alterations of hematological and biochemical markers, the authors reported significant changes in various behavioral parameters, including appetite, weight, activity, and sleep, which are crucial indicators in the determination of acute toxicity. Examination of the excretion profile can provide insight into how the body removes the NFs, which might aid in long-term health studies. After 90 days of monitoring, the hematological and biochemical markers used to indicate potential toxicity were back to the normal ranges, and the behavioral measures returned to usual. Moreover, the terminal histological analysis also showed no sign of inflammation or injury in major organs. However, significant accumulation of QDs was found in the liver, spleen, and kidney after 90 days, suggesting that QDs showed less clearance than biodegradable polymer NPs [222]. Thus, although QDs have low acute toxicity and were thought to be candidates for use in specific clinical applications, their chronic accumulation and potential side effects in the liver, spleen, and kidney cannot be ignored. Generally, nonhuman primates should only be considered for necessary behavior assessments in nanotoxicity studies when no other research model is available to provide the required information.

## 5. Toxicological Endpoints of Observing

### 5.1. In Vitro Cell Viability, Cytotoxicity, and Cell Proliferation

Researchers have most often assessed the viability, cytotoxicity, and proliferation of NF-treated cells as in vitro toxicological parameters [223,224] when seeking to compare the toxicological effects of different NFs or evaluate the toxicity of components used to construct NFs. Both viability and proliferation assays measure the minimum effective concentration (MEC), half-maximal effective concentration (EC_50_), and the maximum effect (E_max_), whereas cytotoxicity assays measure the half-lethal concentration (LC_50_) [225,226]. In general, viability assays monitor the cellular metabolism and enzyme activity and quantify the percentage of living cells in a population, thus reflecting the cytotoxicity of the NF. In contrast, proliferation assays monitor DNA synthesis and cellular division and calculate the number of living cells over time to reveal the genotoxicity of the NF [227]. Both can indicate the dose–response relationship of the treatment, which is crucial for predicting in vivo toxicity and estimating the starting concentrations for in vivo studies.

Monitoring the endogenous biomarkers of cells is a widely used approach to calculate the above parameters. For example, ATP is a useful marker that can be applied to estimate the viability or proliferation of mammalian cells, as its concentration reflects the metabolic activity of viable cells [228,229]. A luminescence-based ATP assay has been developed as a high-throughput test with a <10 min peaking time and >5 h half-life of quenching for the luminescent signals. An ATP assay is usually a terminal assay, requiring cell lysis. Moreover, proper negative and positive controls should be carefully selected and included in the test for validating the assay. Enzymes can also be regarded as endogenous biomarkers; their detection often requires substrates with a light-emitting functional group. The lactate dehydrogenase (LDH) assay measures the extracellular concentration of LDH, which indicates the extent of cell membrane damage following apoptosis or necrosis [230]. The LDH concentration is correlated with the nicotinamide adenine dinucleotide/nicotinamide adenine dinucleotide hydrogen (NAD^+^/NADH) redox circle, which converts yellow tetrazolium salt to blue formazan and can be quantitated using a spectrophotometer.

Many other non-biomarker-based methods have been developed to measure cell metabolic functions, such as those that rely on intracellular dehydrogenase enzymes. Normal living cells can efficiently convert yellow tetrazolium salts to dark blue formazan via the NAD^+^/NADH redox cycle, which is involved in the respiratory chain and catalyzed by mitochondrial succinic dehydrogenases (SDH). NFs that impair mitochondrial membrane structure or respiratory activities could affect SDH function and thus can be indirectly detected by this color change [231]. There are two types of tetrazolium salts: The first type comprises the cationic salts (such as methyl-thiazolyl-diphenyl-tetrazolium bromide (MTT)), which can be readily absorbed by the cell via electrostatic interactions. In this case, NADH converts MTT to water-insoluble MTT-formazan, which can be dissolved in DMSO and quantitated by spectrophotometry [232]. The second type comprises the anionic tetrazolium salts, which include 3-(4,5-dimethylthiazol-2-yl)-5-(3-carboxymethoxyphenyl)-2-(4-sulfophenyl)-2H-tetrazolium inner salt (MTS), 4-(3-(4-iodophenyl)-2-(4-nitro-phenyl)-2H-5-tetrazolio)-1,3-benzene sulfonate (WST-1), and 2,3-bis(2-methoxy-4-nitro-5-sulfophenyl)-2*H*-tetrazolium-5-carboxanilide inner salt (XTT). MTS, WST-1, and XTT are negatively charged (electron donors) and thus are not prone to penetrate the cell membrane. With the help of electron-coupling reagents (electron acceptors, such as phenazine methosulfate or phenazine ethosulfate), MTS, WST-1, or XTT will be taken up by living cells and converted to water-soluble formazan, which can be quantified by a scanning spectrophotometer [233]. These tetrazolium reduction assays are widely used to directly reveal the stage of cell viability/cytotoxicity or indirectly indicate the cell proliferation activity. However, they are toxic to cells and are mostly considered to be terminal measurements.

Similar to tetrazolium salt, resazurin sodium salt (AlamarBlue) is an indicator of metabolically active cells [234,235]. The reduction of resazurin sodium decreases the blue color and concomitantly increases the red fluorescence formed by the intermediate, resorufin. This reaction can be used to monitor metabolic enzyme activity and indirectly evaluate the proliferation of living cells. Cytoplasmic esterase can also be used to indicate cell viability; it hydrolyzes hydrophobic ester to hydrophilic acid, alcohol, or phenol. Calcein acetoxymethyl ester (Calcein-AM) is a rapidly hydrolyzed dye that acts as a substrate of intracellular esterase [236]. Calcein-AM releases intensely fluorescent hydrolyzed calcein inside a living cell, and the intracellular esterase concentration can be quantitated by spectrophotometry. Unlike these above assays, trypan blue stain measures the number of dead cells, which have damaged cell membranes that cannot extrude the blue dye outside the cells [237]. Trypan blue staining can be easily examined under a microscope or counted using a hemocytometer. However, this puts such assays at risk for human errors in counting and difficulties in differentiating between the dead cells and viable cells that are incapable of excluding blue dye.

### 5.2. Detecting the Oxidative Stress and Inflammation

Excessive ROS and reactive nitrogen species (RNS) are the major origins of oxidative stress [238], and there are several types of detection approaches have been developed.

#### 5.2.1. ROS/RNS Detection

The first type of approach directly measures the cellular or extracellular concentration of ROS/RNS. 2′,7′-Dichlorohydrofluoreiscen diacetate (H_2_DCFDA) is a cell-permeable fluorogenic probe for the detection of overall oxidative stress [239,240]. Cell-internalized H_2_DCFDA can be hydrolyzed by cytosolic esterase to release DCFH, which is then oxidized by ROS/RNS to generate fluorescence-emitting 2′,7′-dichlorofluorescein (DCF). DCF fluorescence can be easily measured by various strategies, such as fluorescence spectrometry, flow cytometry, and fluorescence microscopy; thus, this method is widely used. One limitation is that the DCFH-to-DCF conversion is nonspecific and does not differentiate among specific radical species. A recently developed electrochemical method solved this problem by using bimetallic Pd/Au thin-film surfaces for the differentiation of ROS and RNS [241]. The authors integrated two different metallic species (Pd and Au) into a probe and established a sensitive method for the simultaneous detection of H_2_O_2_ ROS and 3-NT (a type of RNS). This method has opened a window for detecting a broad range of both redox and non-redox active species with high sensitivity and selectivity.

#### 5.2.2. Detecting the Oxidative Stress-Induced Damages

The second type of approach is to detect the damages caused by oxidative stress. RONS may cause damage to various biomolecules, including lipids, proteins, carbohydrates, nucleic acids, and cellular metabolites. Measuring these end products is a widely accepted strategy for detecting oxidative stress. For example, monitoring of 2-thiobarbituric acid (TBA) has been used to measure reactive aldehyde malondialdehydes (MDAs), which are the major oxidative products of lipid peroxidation [51,61,242]. Following the addition of TBA to the processed samples, TBA-MDA adducts can be quantitated by fluorescence spectrometry (at 532 nm) or by high-performance liquid chromatography (HPLC); this has proven to be a very sensitive quantitation method. In the context of protein oxidation, carbonylation is a well-characterized process that oxidizes exposed amine groups on several amino-acid residues (e.g., arginine, histidine, lysine, proline, or threonine) and generates protein carbonyls. Carbonyls with adjacent aldehydes or ketones groups can be derivatized by 2,4-dinitrophenylhydrazine (DNPH) to form quantifiable hydrazones [243,244,245]. Immunological techniques have also been developed; they use antibody–antigen recognition to detect DNPH-derivative proteins and protein nitration products.

#### 5.2.3. The Oxidative Response System

The third type of detection method involves the monitoring of biodefensive molecules that respond to oxidative stress. Endogenous SOD and reduced GSH respectively represent the first-line enzymatic and nonenzymatic antioxidants in the complex antioxidant defense grid of the biosystem [246]. Substrates of SOD, such as dihydroethidium and nitro-tetrazolium blue, have been used to assess superoxide-dependent SOD activities [247,248]. Under oxidative stress, SOD converts dihydroethidium or nitro-tetrazolium blue into fluorescent end-products that can be quantitated by fluorescence spectrometer. Alternatively, the protein expression of SOD is often evaluated by immunoblotting techniques, such as Western blot analysis. Such techniques can also be used to semiquantify other crucial enzymes in the oxidative response systems, such as catalase and glutathione peroxidase (GPX) [249]. The gene expression levels of these proteins are often investigated by real-time polymerase chain reaction (RT-PCR) [250,251]. For monitoring reduced GSH, the GSH-to-GSSG ratio is a better indicator than the concentration of GSH, as the former reflects the oxidative responses from the GSH–GSSG cycle [252]. The GSH-to-GSSG ratio can be assessed by various colorimetric, fluorescent, or luminescent molecular probes [253,254,255]. Complementary information obtained from GPX expression analysis could provide further insight into the GSH response to oxidative stress.

#### 5.2.4. Detections with the Multi-Omics Platform

Thanks to the rapid development of sophisticated bioanalytic instruments, numerous biomedical databases, and powerful analytic software, bioanalysis has entered an era of so-called “multi-omics” [256]. Powerful tools such as ultraperformance liquid chromatography/high-resolution mass spectrometry (UPLC-HRMS), gas chromatography (GC)–HRMS, and ultrahigh-field nuclear magnetic resonance (NMR) have increasingly been used to investigate complex biomedical specimens [257,258,259]. These technologies can obtain multiple signals in a single test cycle, simultaneously offering quantitative or qualitative information for multiple components. For example, UPLC-HRMS-based proteomics has been widely adopted for studying redox-mediated protein modifications and quantitating the protein carbonyl sites [260,261]. GC-MS or HPLC has a long history of quantitating different types of low-density lipoproteins and oxidative lipid products, including hydroperoxides and aldehydes [262,263,264]. The integration of platforms such as genomics, proteomics, and metabolomics offers great opportunities for revealing the possible toxic effects of NFs from a global perspective.

### 5.3. Cell Monolayer Permeability and Tight Junction Assays

The Caco-2 cell monolayer permeability assay has been widely used to predict the absorption of a formulated drug and can also be used to assess the state of tight junctions [265]. A steep increase in the permeability of a Caco-2 cell monolayer suggests that the formulation is either cytotoxic or harmful to the cell’s tight junctions [265,266]. Therefore, a cell viability test is usually performed before the monolayer permeability assay, and a concentration that has no apparent effect on the viability of Caco-2 cells will be selected to evaluate the effects of the formulation on tight junctions. To perform the latter study, Caco-2 cells are first cultured on a permeable membrane of a transwell insert, which is placed in the plate with the cell culture medium on both the apical and basolateral sides. After 2–3 weeks, Caco-2 cells form a densely populated cell layer and spontaneously differentiate into polarized enterocytes and a monolayer of columnar cells that are coupled together by tight junction protein complexes. NFs and water-soluble fluorescent dye, such as fluorescein isothiocyanate–dextran (FITC-Dextran 70) or lucifer yellow salt buffer solution, can be spiked to the apical side of the medium. At the desired time intervals, a small amount of medium from both sides of the membrane can be measured by fluorescence spectrometry. As FITC-Dextran and lucifer yellow are unable to penetrate the intact monolayers, a significant increase of fluorescence intensity in the basolateral medium suggests that there has been damage to barrier integrity [267]. Transepithelial electrical resistance (TEER) is another electrical tool that may be used to measure Caco-2 monolayer integrity. The Caco-2 monolayer usually generates a TEER of 150–400 Ω·cm^2^, which restricts the diffusion of substances across the barrier [114]. A sharp decrease in the TEER value upon NF treatment suggests that the tested formulation is nanotoxic.

### 5.4. Clonogenic Assay

The clonogenic assay is a technique for assessing the effect of a test formulation on the survival and proliferation abilities of cultured cells [268]. In this assay, cells are plated at a very low density or even a single cell and allowed to grow until visible colonies are formed. Pretreatment with or without the target NFs in the medium can be used to assess the impact of the NF on colony formation. The result can be visualized and quantitated by nuclear stains, such as crystal violet or methylene blue. D. Toomeh et al. applied this technique to evaluate the potential of graphene oxide nanoflakes (GONFs) against cancer recurrence and metastasis [269]. Together with the MTT assay, the clonogenic test confirmed that GONFs effectively inhibited the survival of cancer cells during radiotherapy. Other researchers have used this technique to evaluate the cytotoxicity of Ag NPs against various types of cancer cells, including lung, ovarian, and breast cancer cells [270]. Although this method has not yet been applied in oral nanotoxicity models, it should be a convenient and available technique for such studies.

### 5.5. Genotoxicity

#### 5.5.1. Chromosome Aberration

Chromosomal aberration is a strong indication of genotoxicity [271]. This damage can be directly identified using simple staining techniques (e.g., 5% Giemsa) followed by microscopic analysis [272]. If a chromosomal aberration is identified, it indicates that chromosomes have undergone disruption/breakage due to the presence of a mutagen. Typically, cells are pretreated with cell cycle synchronizing agents such as colcemid or colchicine at predetermined intervals, then treated with or without the NF during S-phase [273]. The presence of micronuclei is also a strong indicator of chromosomal breakage, as they represent chromosomal fragments or whole chromosomes that have not been incorporated into the nuclei of newly formed daughter cells [274]. For assessment of micronuclei, cytochalasin B is typically employed to inhibit cytokinesis, and micronuclei are assessed in binucleated cells.

#### 5.5.2. Single-Cell Gel Electrophoresis (Comet Assay)

Single-cell gel electrophoresis (SCGE), also called the comet assay, is a method for detecting strand damage in the DNA of a cell [275,276]. For this technique, cells are embedded in agarose gel, lysed, and treated with salt, and then the gel is subjected to alkaline electrophoresis. Intact nucleoids are supercoiled DNA; any toxicity-induced breaks present in the nucleoids will relax this supercoiling to form DNA loops. These loops extend toward the anode of the apparatus, forming a “comet tail”. Fluorescent dyes are used to stain and visualize the DNA molecules, and image analysis software can be used to quantify the observations. Improved comet assays can be used to evaluate DNA damage in various cell types, including those of yeast, protozoa, plants, invertebrates, and mammals, thus offering an excellent platform for evaluating nanotoxicity-induced gene damage [277].

#### 5.5.3. Histochemical Approach

The traditional method of identifying cell proliferation is by microscopically observing and counting cells. By such a method, mitotic progression can be observed in the presence of experimental NFs. The result is shown as the “mitotic index”, which represents the number of mitotic cells divided by the total number of observed cells [278]. Measurement of the mitotic index also requires the addition of the cycle synchronizing agents (e.g., colcemid or colchicine) to arrest cells in metaphase, so that cells undergoing mitosis can be easily identified. Another approach is to monitor cell proliferation by measuring the incorporation of [^3^H] thymidine into new strands of chromosomal DNA during mitotic cell division [279]. However, this method has various limitations, such as incomplete uptake of the isotope label, the requirement of a scintillation counter to detect the label, and a long incubation period of 24–48 h. An alternative option is to incorporate bromodeoxyuridine (BrdU) and use antibodies and flow cytometric analysis to detect incorporated BrdU [280,281]. BrdU is more specific than [^3^H] thymidine to cells undergoing DNA synthesis.

Toxicity-induced DNA fragmentation and chromatin condensation can be captured by the terminal dUTP-transferase-mediated nick end labeling (TUNEL) assay [282] or staining with Apostain, which is a DNA dye that can penetrate the plasma membrane [283,284]. In TUNEL assay, the ends of DNA fragments are labeled at their 3’-OH ends by biotinylated dUTP; this labeling is detected using streptavidin–horseradish peroxidase and a diaminobenzidine chromogen and visualized under light microscopy. Alternatively, the dUTP nucleotides can be labeled with a fluorescent dye and visualized by fluorescent microscopy. The Apostain assay is more accurate as it stains condensed chromatin: the nuclei are first denatured by heating in the presence of MgCl_2_, then the single-stranded DNA of condensed chromatin from denatured nuclei is targeted using Apostain antibodies.

#### 5.5.4. Immunohistochemical Approach

The antigen, Ki67, is a nuclear protein that is present during the active phases of the cell cycle (G1, S, G2, and M) but absent from the resting state (G0 phase). It can be used as a cell proliferation marker, as a significant increase of Ki67 protein (pKi67) indicates active cell proliferation [285,286]. pKi67 is well characterized at the molecular level and can be used to indicate relative levels of cell proliferation activity. Proliferating cell nuclear antigen (PCNA) is a homotrimer complex that serves as a DNA clamp involved in DNA synthesis and repair [287,288]. The presence of PCNA during the early G1 and S phases of the cell cycle is strongly associated with cell proliferation and can be used to assess the effects/toxicity of NFs.

#### 5.5.5. Measurements of the Gene Expression Changes

Gene expression changes have often been assessed by traditional methods, such as Northern blot analysis or ribonuclease protection assays (RPAs) [289,290]. Northern blot analysis is a standardized method for both quantitation and detection of mRNAs. The assay provides a relative comparison between samples on a membrane and is a preferred method for detecting the presence and sizes of alternatively spliced transcripts. RPA is used to detect and quantify specific mRNA transcripts from a mixture of RNA molecules. In this process, a synthetic RNA probe that is complementary to the target of interest is engineered. A radioactively labeled probe can also be incorporated into the design. Then, ribonuclease is applied to the mixture of single-stranded RNA and double-stranded probe–target hybrid. Once the single-stranded RNA is digested, the sample is electrophoresed on a denaturing Tris/Borate/EDTA (TBE)–urea polyacrylamide gel, and the labeled probes are visualized.

Another widely used method is real-time polymerase chain reaction (RT-PCR), which is a quantitative method for determining the number of copies of PCR templates like DNA or cDNA [291]. RT-PCR methods can be probe-based or intercalator-based. In addition to a pair of primers, probe-based RT-PCR requires a fluorogenic oligonucleotide such as the TaqMan probe, which has both a reporter fluorescent dye and an attached quencher dye. Intercalator-based RT-PCR often uses the SYBR green fluorescent dye, which binds to double-stranded DNA. Both methods employ instruments equipped with a thermocycler and require a special camera to monitor the fluorescence in each well of the plate.

Microarray analysis is a high-throughput method that can be used to measure the mRNA levels of a cell. It employs microchips coated with arrays of short DNA probes and can be used for large-scale screening [292]. Samples such as cell or tissue lysates are subjected to RNA extraction, and the RNA is converted to cDNA with fluorescent tags or radioactive labels. These labeled cDNAs are incubated with the chip, which is then washed and scanned using a laser scanner or autoradiographic imager. The obtained data are compared with those in a relevant database and then further analyzed. Microarray analyses can reveal transcriptomics information following NF treatment and thereby contribute to a holistic understanding of the cellular responses that cannot be achieved by the standard in vitro assays.

#### 5.5.6. Measuring Oxidized Guanosine

Guanine has the lowest reduction potential among the four DNA bases and is the most readily oxidized base [293]. Under oxidative injury, guanosine is oxidized to 8-oxo-deoxyguanosine (8-Oxo-dG), and its corresponding base, guanine, is oxidized to 8-oxo-guanine (8-Oxo-Gua) [294]. This process usually induces point mutations within a gene during DNA replication. For example, incorporation of adenine opposite 8-Oxo-dG induces the G:C→T:A transversion. 8-Oxo-dG and 8-Oxo-Gua can also be further oxidized, causing more complicated oxidative lesions. 8-Oxo-dG and 8-Oxo-Gua can be measured using immunohistochemistry, HPLC-electrochemical detection, or LC-MS/MS methods. In animal studies and clinical tests, urinal 8-Oxo-dG and 8-Oxo-Gua may be quantitated as an indicator of DNA oxidative damage [295].

### 5.6. Method Validation

Most of the standardized toxicity assays were developed for evaluating conventional drugs or formulations [224,296]. Although these assays can be applied to assess the toxicity of NFs, they require modifications and method validation. For example, NFs may interfere with conventional detection methods based on the optical properties of molecular probes (e.g., absorbance, fluorescence, or luminescence) by scattering or absorbing the light within the monitored spectral range [297,298]. More specifically, Au- and Ag-NPs show absorption peaks within the same spectral range used by several molecular probes, obstructing the absorbance readings and reducing the fluorescence signals. Other NFs, including carbon nanotubes and superparamagnetic iron oxide (Fe_3_O_4_) NPs, were found to quench the fluorescence of the probe H_2_DCFDA [299,300], causing miscalculation of RONS concentrations. In addition to hindering optical signals, NFs can directly interact with enzymes and prevent their proper measurement. For example, carbon nanotubes were found to bind to cytokines and prevent their protein–antibody recognition by ELISA [301]. Similar NP-cytokine binding was observed in the testing of dendrimer nanocarriers [302]. Sometimes, NFs can catalyze reactions that alter the detection results: Several metal-based NFs were found to catalyze redox reactions, thus yielding false-positive results in the fluorescence quantitation of formazan [303], whereas Ag NPs, Cu NPs, and ions were found to inhibit LDH activity, giving false-negative data on the LDH assay [304]. For these assays, researchers should add a negative control with empty NFs that will undergo the same assay processing. If using the NFs hinders the optical readings, a sample processing step should be used to remove the NFs before the sample is mixed with the optical probe. Additionally, multiple complementary detection methods (based on different detection mechanisms) should be employed, as this can help avoid systemic errors in the testing of nanotoxicity.

## 6. Strategies for Making Nontoxic Oral NFs

The toxicity of NFs can be built up in many ways; the general rule for avoiding nanotoxicity is to reduce the drug dosage by using targeted NP drug delivery systems and make major efforts to avoid using nondegradable NFs [140]. Along with the rapid development of nanotechnology, this has been facilitated by the development of various biocompatible NFs for drug delivery. Some are commercialized, such as liposomal NPs, iron dextran colloid, and albumin-bonded NFs. However, most of these platforms are dosed via the intravenous route [305]. Oral delivery of NFs is still a challenge, as such NFs must undergo the harsh environment in the GI tract and release the drug to the intended target [306,307].

**Figure 3:**
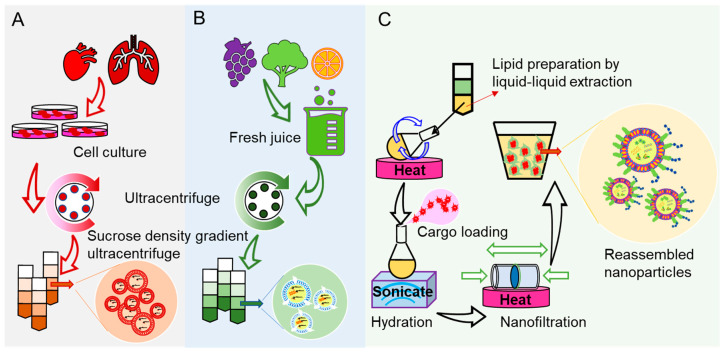
Strategies for making nontoxic oral nanoformulations (NFs): (**A**) preparation of mammalian cell-derived exosomes; (**B**) isolation of plant-derived nanoparticles; (**C**) engineering natural-derived nanoparticles from extracted lipids. *Nanomaterials*. 2020, 10, 1424; DOI:10.3390/nano10071424 [140].

Some formulation strategies have shown promising results in their discovery stages. One such strategy is the use of mammalian cell-secreted exosomes [38,77,308] (Figure 3A). For example, intestinal cell-derived exosomes have demonstrated GI tract stability and colon-targeting ability and thus can be used for drug delivery systems. These exosomes are even stably present in feces. Another strategy is to make use of plant-derived lipid NPs (Figure 3B,C). Commonly consumed fruits, vegetables, and spices have been found to contain a large number of lipid NPs [17,40,309,310,311]. These lipid NPs offer safe nanoplatforms for the oral delivery of drugs, as their components are daily nutrients. For size-controllable synthetic polymers, surface modifications can improve their targeting ability and GI tract stability. Modifications such as surface coatings of PEG, PLA, or PLGA can form stealth NPs that are protected from digestion and too-rapid clearance [312,313].

In cases where the use of metal, inorganic, carbon nanotube, graphene, and other exogenously sourced NFs cannot be avoided, surface modifications are necessary to prevent direct contact at the nano–bio interface [313,314,315,316]. Such materials should also be further purified to remove any residual hazardous materials or catalysts. Such contaminations can trigger inflammatory signaling mediators leading to immune responses. Indeed, the presence of hazardous contaminants or catalysts has been shown to affect the safety of carbon nanotubes [317,318]. The size of an NF is also crucial for safety-related issues: macrophages are believed to rapidly eliminate ultrafine NFs (less than 100 nm) [319,320,321], but the undegradable nature of these NFs may induce their accumulation inside macrophages and cause immune response-related inflammation.

## 7. Conclusions and Prospects

Unlike other delivery routes, oral administration of NFs increases their exposure to widely diversified biological and chemical conditions, including enzymatic- and microbiota-related digestion and ionic strength and pH variations. The harsh conditions of the GI tract challenge the stability of NFs. Nanotoxicity typically originates from the instability of NFs and their unique physicochemical properties. The aggregation and dissociation of NFs in luminal fluids and at the mucus–epithelial cell interface also affect their topical toxicology behaviors.

Researchers urgently need to establish a combination of complementary models for assessing oral nanotoxicity. Although in vivo and ex vivo models can provide useful toxicity information related to human cells and tissues, these models cannot offer accurate information on the absorption, distribution, metabolism, and excretion (ADME) fate of NFs, nor can they provide drug metabolism and pharmacokinetics (DMPK) profiles of the NFs in the host. At present, we still require in vivo models, despite the booming of new animal-replacing organ-on-a-chip and microfluidic technologies.

Enormous efforts have been made to establish detection methods for evaluating the safety of emerging nanomaterials. Many of these methods are derived from the protocols for assessing conventional formulations and thus are not explicitly designed for evaluating nanotoxicity. Given that NFs interfere with optical-based detections in many ways, unmodified methods will undoubtedly lead to inconsistent results. Instead, NFs should be removed before samples are processed for optical-based quantitation, with the goal of minimizing such interference. An obvious yet underappreciated issue is that most of the applied methods lack a stringent method validation step. This is a normal phenomenon in the biomedical field, as many biomedical researchers are more familiar with ready-to-use protocols. Validating a newly established method tends to fall more within the research interests of analytical chemists or analytical biochemists.

Nanotoxicology is still a developing discipline. New technique-based platforms (such as mass-spectrometry-based proteomics and microarray-based transcriptomics) are powerful tools for investigating toxic responses of the cells or tissues at a global level, providing comprehensive information that cannot be obtained with traditional probe- and antibody-based assays. These platforms can also largely bypass the NFs’ optical interference issues: the hyphenated detection techniques, such as LC-MS or capillary electrophoresis–mass spectrometry (CE-MS), are equipped with strong separation capabilities and are built for multi-omics studies that tackle samples within a complex biomatrix. Going forward, nanotoxicology studies can make full use of these new techniques, which will open our minds to designing better toxicology models for studying nanotoxicity.
